# Field Research Is Essential to Counter Virological Threats

**DOI:** 10.1128/jvi.00544-23

**Published:** 2023-05-11

**Authors:** Jonathan A. Runstadler, Anice C. Lowen, Ghazi Kayali, S. Mark Tompkins, Randy A. Albrecht, Ron A. M. Fouchier, David E. Stallknecht, Seema S. Lakdawala, Felicia D. Goodrum, Arturo Casadevall, Lynn W. Enquist, James C. Alwine, Michael J. Imperiale, Stacey Schultz-Cherry, Richard J. Webby

**Affiliations:** a Department of Infectious Disease and Global Health, Cummings School of Veterinary Medicine, Tufts University, Boston, Massachusetts, USA; b Center for Research on Influenza Pathogenesis and Transmission (CRIPT) CEIRR, Icahn School of Medicine at Mount Sinai, New York, New York, USA; c Department of Microbiology and Immunology, Emory University School of Medicine, Atlanta, Georgia, USA; d Emory Center of Excellence for Influenza Research and Response (Emory-CEIRR), Atlanta, Georgia, USA; e Human-Link DMCC, Dubai, UAE; f University of Texas School of Public Health, Houston, Texas, USA; g St Jude Center of Excellence for Influenza Research and Response (SJ-CEIRR), Memphis, Tennessee, USA; h Center for Vaccines and Immunology, University of Georgia, Athens, Georgia, USA; i Center for Influenza Disease and Emergence Research (CIDER) CEIRR, Athens, Georgia, USA; j Department of Microbiology, Icahn School of Medicine at Mount Sinai, New York, New York, USA; k Global Health and Emerging Pathogens Institute, Icahn School of Medicine at Mount Sinai, New York, New York, USA; l Department of Viroscience, Erasmus Medical Center, Rotterdam, Netherlands; m Southeastern Cooperative Wildlife Disease Study, College of Veterinary Medicine, University of Georgia, Athens, Georgia, USA; n Penn Center of Excellence for Influenza Research and Response (Penn-CEIRR), Philadelphia, Pennsylvania, USA; o Department of Immunobiology, University of Arizona, Tucson, Arizona, USA; p Department of Molecular Microbiology and Immunology, Johns Hopkins School of Public Health, Baltimore, Maryland, USA; q Department of Molecular Biology, Princeton University, Princeton, New Jersey, USA; r Department of Cancer Biology, Perelman School of Medicine, University of Pennsylvania, Philadelphia, Pennsylvania, USA; s Department of Microbiology and Immunology, University of Michigan, Ann Arbor, Michigan, USA; t Department of Infectious Diseases, St Jude Children's Research Hospital, Memphis, Tennessee, USA; University of North Carolina at Chapel Hill

**Keywords:** Field research, Pandemic preparedness, Risk assessment, Spillover, Surveillance, Virology, Wildlife, Zoonosis

## Abstract

The interface between humans and wildlife is changing and, with it, the potential for pathogen introduction into humans has increased. Avian influenza is a prominent example, with an ongoing outbreak showing the unprecedented expansion of both geographic and host ranges. Research in the field is essential to understand this and other zoonotic threats. Only by monitoring dynamic viral populations and defining their biology *in situ* can we gather the information needed to ensure effective pandemic preparation.

## TEXT

Viral spillovers have been prominently featured in recent news articles, commentaries, and health reports (1, 2). Much of this attention has been driven by the global spread of the H5 subtype highly pathogenic avian influenza virus (HPAIV). Spanning multiple continents, this epizootic has had an enormous ecological impact, owing to the widespread mortality in dozens of wild bird species (3, 4). Furthermore, spillover into poultry holdings across broad geographical areas has necessitated the destruction of millions of domestic birds, which impacted the poultry industry in many regions and threatened food security (3, 5, 6). Concerningly, the infection of more than 30 mammalian species with HPAIV has been documented in recent months, with large die-offs suggesting that marine mammals are being heavily affected (3, 7). While the probable transmission of the virus between mammals has been reported only once (8), the epidemiology of marine mammal outbreaks (9, 10) and the high frequency of isolated cases in mammals raise concern. Furthermore, experimental work has convincingly demonstrated that HPAIV has the capacity for mammalian transmission (11, 12), with studies having alerted humanity of this threat more than 10 years ago. Spillover into mammals creates opportunities for viral adaptation to a mammalian host in nature (13, 14), which may, in turn, increase the risk to humans.

The scope and character of the present HPAIV outbreak are alarming. These viruses present multiple transmission pathways to both domestic animals and humans, and they continue to rapidly change. Surveillance and hypothesis-driven field research are both critically needed to define geographical reach, detect host range expansion, and track viral evolution. Unfortunately, this outbreak comes at a time when resources for monitoring influenza viruses in wildlife are scarce. After a period of healthy investment from United States national funding agencies, support has faltered in recent years. As an example, the National Institutes of Health (NIH) Centers of Excellence for Influenza Research and Surveillance (CEIRS) program, which was initiated in 2007, recognized the direct connection of viral ecology and evolution in nonhuman hosts to pandemic preparedness and built a valuable infrastructure with which to address global and sometimes urgent challenges. When the CEIRS program was continued by the Centers of Excellence for Influenza Research and Response (CEIRR) program, which was initiated in 2021, it took a major step away from field studies in wildlife hosts. While there are likely multiple drivers of this change in research priorities, a troubling contributor is the failure to recognize that a sustained effort with a global reach that spans a large swath of viruses and their hosts is absolutely essential to detect, assess, and define risks early as well as intervene.

**FIG 1:**
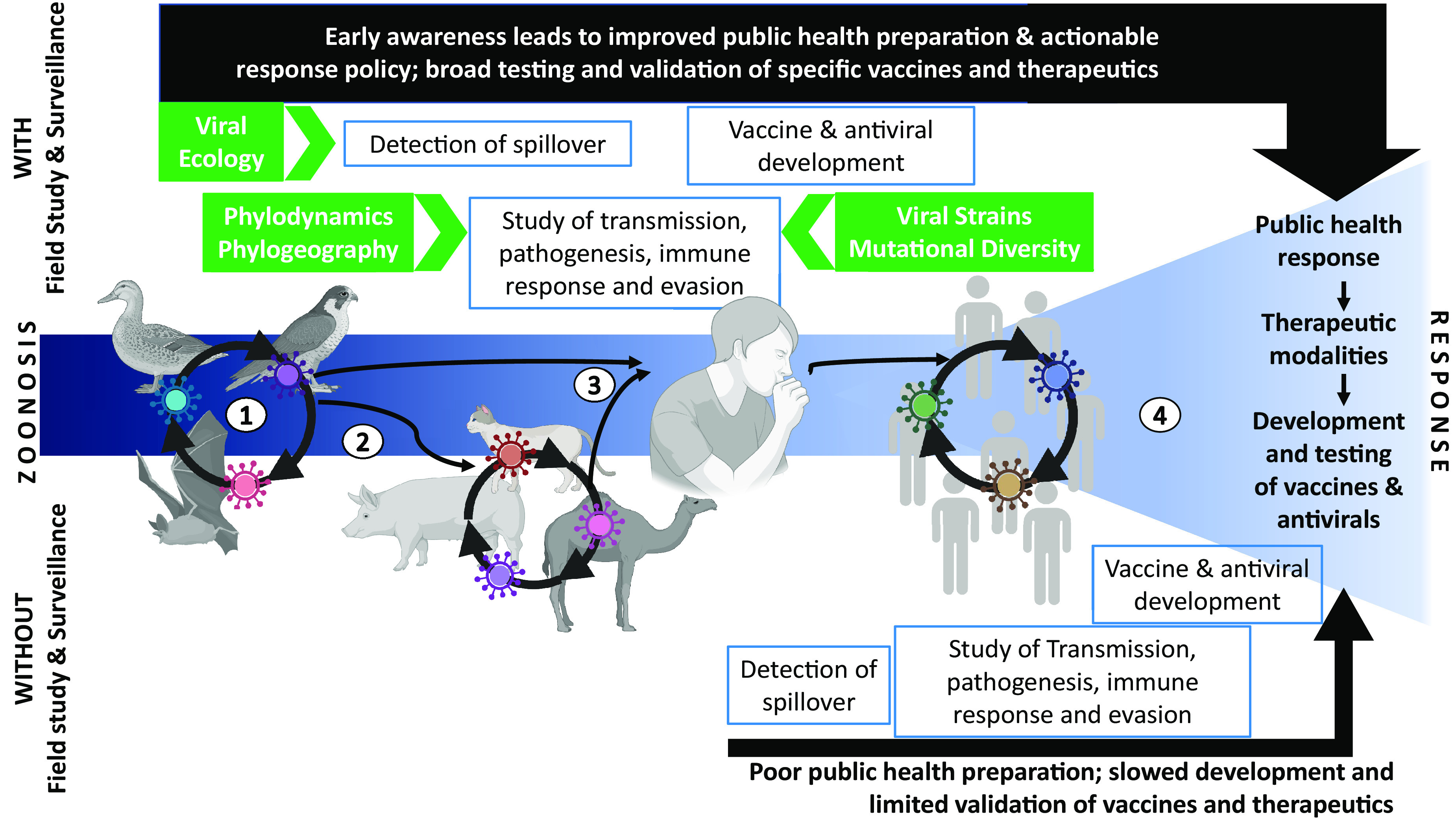
Expanding the timeline for strong pandemic preparedness. Schematic illustrating the sequence of a zoonotic spillover event that results in human-to-human spread as well as the pandemic response with (top) or without (bottom) prior field work and surveillance. The timeline for zoonosis: (i) intraspecies and interspecies spread, (ii) jump to a new species or intermediate host, (iii) jump to humans, and (iv) human-to-human transmission. The back linear arrows (top and bottom) indicate the timeline for the accumulation of data and knowledge for an informed pandemic response in each scenario. The weight of the arrows indicates the strength of the response. The green boxes and arrows indicate the knowledge and viral isolates that were obtained via surveillance to inform and fortify the response to pandemic threats. Field data that are collected as well as surveillance work that is conducted prior to detection of the jump into and spread between humans will shorten the time frame of the response. In this way, the knowledge, viral isolates, and data that are required to understand transmission, pathogenesis, and immune responses are accumulated prior to sustained transmission in humans. This knowledge will, in turn, support improved public health preparation through modeling, the development of actionable response policies, and the validation of novel vaccine and therapeutic modalities. Furthermore, the knowledge gained through field research can enable risk mitigation to be implemented in nonhuman populations (at steps ii and iii) with a much smaller societal impact, compared to mitigations that are designed to prevent the transition from step iii to step iv. In the absence of robust surveillance infrastructure, the response is slower, weaker, and more likely to impose high societal and economic costs.

The majority of emerging and reemerging viruses arise from wildlife species (Table 1). However, the mechanisms and interactions leading to emergence are poorly understood. For example, for influenza A virus, which is relatively well-studied, the evolutionary changes needed to support viral expansion into a new host species are incompletely understood. For other potentially zoonotic pathogens, this understanding is lacking almost entirely. The ecological context in which such evolutionary change is likely to occur is another major knowledge gap. For instance, do intermediate hosts drive viral evolution that increases human risk, or does such evolution occur during low level circulation in humans as a necessary precursor to a wider outbreak? Without insight into these and related questions, options to prepare for or prevent a pandemic are limited. We do currently understand that ecological disturbances and the anthropogenic interface play major roles in driving emergence. Major risk factors include land use changes (including farming practices), climate fluctuations, and habitat encroachment (15). Public health actions can help control emerging diseases but these actions are reliant on field studies that are designed to detect and understand both long-term and short-term risks. Transient or geographically limited surveillance efforts are not well-suited to meet this challenge (Fig. 1).

**TABLE 1:** Major viral zoonotic outbreaks of the past 50 years

Pathogen	Year(s) of outbreak(s)	Proximal source	Reservoir host
Mpox virus	2022	Unknown	Rodents
SARS-CoV-2	2019, ongoing	Unknown	Bats
Zika virus	2015 to 2017	*Aedes* species mosquitos	Nonhuman primates
Ebola virus	2014 major, multinational outbreak; 1976 to present, sporadic outbreaks	Often bushmeat	Unknown
H7N9 influenza virus	2013 to 2017	Poultry	Wild waterfowl
MERS-CoV	2012	Camels	Bats
H1N1 influenza virus	2009, ongoing	Swine	Wild waterfowl^*a*^
SARS-CoV	2003	Civet cats	Bats
West Nile virus	2002 to 2003	*Culex* species mosquitos	Birds
Nipah virus	1999 to present, sporadic outbreaks	Swine/bats	Fruit bats
H5N1 influenza virus	1997, 2003, ongoing	Poultry	Wild waterfowl
HIV	1981, ongoing	Unknown	Nonhuman primates
Marburg virus	1967 to present, sporadic outbreaks	Often bushmeat	Egyptian rousette bat, others?

a The H1N1 subtype influenza A virus that caused the 2009 pandemic carried gene segments from viral lineages that were circulating in multiple distinct host reservoirs (swine, human, and avian). Each of these lineages is ultimately derived from those that were circulating in wild waterfowl.

While the comprehensive sampling of viruses that pose a potential threat is a lofty goal, the evidence-based prioritization of field research efforts can be used to focus resources. Studies of the ecological and molecular processes that drive the evolution, persistence, and circulation of novel viruses can reveal host species and ecological conditions that increase risk. Field studies directed at such viral reservoirs can then yield testable hypotheses about the likelihood of changed exposure routes or viral evolution leading to human outbreaks. When surveillance and field research are well-integrated with laboratory-based studies on viral pathogenesis and transmission, these hypotheses feed efforts to anticipate viral emergence and develop responses. Such advances lead to improved pandemic preparedness (Fig. 1).

Too often, the scope of experimental virology is limited by a lack of integration with field work. Without field research, the risk assessment for pandemic preparedness is likely to be underpowered. Studies in disease ecology and virus natural history identify new lineages and viral strains and offer the epidemiological data that are needed to test hypotheses about priority viruses with pandemic potential. At the most basic level, field research also supplies viral isolates and sequence data that are critical for downstream risk assessment. In the United States, proposed field studies undergo review by institutional oversight committees that govern the use and care of animals (Institutional Animal Use and Care Committee [IACUC]) as well as biosafety (Institutional Biosafety Committee [IBC]), including any proposed interactions with wildlife that are the subjects of field research. Research teams are trained and advised on the prevention of potential zoonotic infections, including vaccination, where appropriate, as well as the use of personal protective equipment (PPE) to prevent exposure. In many countries, these studies require involvement, permission, and oversight by local governmental bodies, including Ministries of Health, Agriculture, or Environment. All field sites are unique, but awareness, training, and preparation ensure that in each case, samples and personnel are protected to minimize risk that is far outweighed by maximizing the integrity and use of the information that is gained to protect public health. It is essential for our efforts to combat viral threats that the link between pandemic preparedness and studies of virus ecology and evolution in reservoir hosts be recognized by the virology community, by funders, and by the public.

In addition to providing essential information and materials for downstream virological research, field research often directly informs public health policy. The ongoing HPAIV outbreak offers an instructive example. Based on rapid investigations in the field, decisions are being made in real-time about how to restrict public access to wildlife areas, how animals are housed and handled in businesses, and how animal carcasses in the field are disposed of. In addition, interventions to cull infected animals reduce opportunities for human transmission (16). Concomitantly, the informed development and testing of therapies and preventive measures rely on field efforts that are designed to capture the breadth of diversity of contemporary viruses and to furnish representative isolates. The information from field studies is directly applied to the selection of candidate vaccine viruses for pandemic preparedness and stockpiling, for the assessment of approved and experimental antiviral drugs and therapeutics, and to provide goals for the assessment of next-generation vaccines. The urgency with which data from the field are needed to inform policy in an outbreak situation is important to consider in light of a recent recommendation by the National Security Advisory Board on Biosafety (NSABB) to extend the oversight of the dual-use research of concern (DURC) to include surveillance activities (17). The administrative burden that this oversight would impose could paralyze effective field efforts on major zoonoses (18).

Furthermore, field studies on potential zoonotic viruses are central to the One Health paradigm, which highlights the interconnectedness of animal, human, and environmental health. Viruses that circulate in animals that are raised for food can present transmission risks to humans and can also threaten food supplies. Viruses of wildlife are similarly of concern when they cross species barriers to humans or domestic animals, and they are also an important consideration in environmental health. In the case of the current H5N1 outbreak, a virus that emerged in poultry is wreaking havoc on wild animal populations. 10 years ago, it was estimated that there were more than 300,000 mammalian viruses that remained to be discovered (19). This is a number that is almost certainly an underestimate, based on what we have learned in the past decade about viral diversity. Virology that starts in the field can identify specific host species, natural history, and environmental conditions that increase the risks of transmission to other wildlife species, domestic animals, and humans, all of which are linked through interfaces in our shared environment, activities, and industry.

Viral emergence events are expected to occur more frequently due to the expanding human overlap of wild and domestic animal reservoirs and because such reservoir populations and the viruses that they carry are constantly changing. To address this threat, humanity needs to deploy active and sustained viral surveillance as well as field research across the globe. Together with complementary experimental studies, these efforts will strengthen our abilities to identify and respond to pandemic threats before they achieve sustained human-to-human transmission.
